# Therapeutic plasma exchange in acute fatty liver of pregnancy: a case report and literature review

**DOI:** 10.11604/pamj.2021.40.220.31324

**Published:** 2021-12-13

**Authors:** Ajay Kumar, Anand Sharma, Yatendra Mohan, Itish Patnaik, Ashok Kumar, Kavita Khoiwal, Gita Negi, Rohit Gupta

**Affiliations:** 1 India Institute of Medical Sciences, Rishikesh, India

**Keywords:** Acute liver failure, jaundice complicating, pregnancy, herapeutic plasma exchange, case report

## Abstract

Acute fatty liver of pregnancy (AFLP) is characterised by acute liver failure that occurs most commonly in the third trimester of pregnancy. Emergent delivery of the foetus reverses liver failure in most cases. Rarely, termination of pregnancy may not reverse liver failure, and adjunct interventions may be required. Therapeutic plasma exchange (TPE) has been described in AFLP in very few reports. We describe a patient in whom liver failure and extrahepatic organ failure persisted four days after delivery. She underwent TPE for persistent liver failure which resulted in prompt clinical improvement. We propose that TPE be considered as a measure to salvage AFLP patients with liver failure that does not reverse after termination of pregnancy.

## Introduction

Acute fatty liver of pregnancy (AFLP) is a rare but potentially life-threatening hepatopathy seen in the later stages of pregnancy, the hallmark of which is liver failure. Prompt delivery of the foetus results in recovery of liver function in most cases. Therapeutic plasma exchange (TPE) has been described in few reports of AFLP to reverse liver failure that does not respond to termination of pregnancy alone. We describe a lady with AFLP who continued to have liver failure despite termination of pregnancy. She underwent therapeutic plasma exchange (TPE) that resulted in prompt clinical recovery. This intervention may help salvage the rare instances in this rare disease that do not respond to the delivery of the fetus and prevent maternal mortality, especially in a resource-constrained setting.

## Patient and observation

**Patient information**: a 22-year-old primigravida presented to the emergency department with yellow discolouration of eyes and urine and recurrent non-bilious vomiting for five days at 36 weeks of hitherto uneventful gestation. She did not experience fever, aches, joint pains, itching, pale stools, abdominal pain, abdominal distension, constipation, reduced urine output, headache, altered sensorium or vision. She was not on any drug known to cause liver injury. Her past or family history was unremarkable for liver-related illness. She was normotensive and regularly vaccinated during her antenatal follow-up.

**Clinical findings**: the patient was afebrile, drowsy and had icterus and flapping tremors. Her blood pressure was 110/70 mmHg, Pulse rate was 103 per minute, SpO_2_ was 95%, and respiratory rate was 24 breaths per minute. Abdominal examination revealed a gravid uterus and no hepatosplenomegaly or dilated veins over abdominal wall.

**Diagnostic assessment**: laboratory investigations at admission (Table 1) revealed leucocytosis, thrombocytopenia, features of liver dysfunction (hyperbilirubinemia, transaminasemia, coagulopathy) and acute kidney injury(AKI). Arterial blood gas analysis was normal. Urine dipstick for protein was negative. Ultrasound abdomen revealed fatty liver, ascites with no features of chronic liver disease. Ascitic fluid analysis showed high serum albumin ascitic fluid gradient (SAAG) with no evidence of peritonitis.

**Table 1:** laboratory investigations during the course of illness

Variables	On Admission	Postpartum day 1	Postpartum day 4 (Before 1^st^ TPE session)	Postpartum day 5 (Before 2^nd^ TPE session)	Postpartum day 6 (Before 3^rd^ TPE session)	Post-partum day 7	Post-partum day 9	Post-partum day 15
Haemoglobin (g/dl)	13.6	7.3	6.7	7.7	8.1	8.8	9.3	8.7
Total leucocyte count (percumm)	15.9	25.1	22.5	14.2	13.8	15.6	10.1	6.5
Platelets (per cumm)	1,00,000	45,000	45,000	31,000	50,000	52,000	50,000	2,10,000
Urea (mg/dl)	28.7	47	64	53	47	48		18
Creatinine (mg/dl)			1.6	0.7	0.6	0.7		0.4
International normalised ratio	2.7	2.9	3.0	1.3	1.3	1.1	1.1	1.2
Total Bilirubin (mg/dl)	12.5		11.8	12.6	10.5	8.5	8.4	8.9
Direct bilirubin (mg/dl)	9.3		8.7	7.0	6.2	5.3	5.8	5.3
Aspartate aminotransferase (IU/L)	315		77	57	45	52	62	60
Alanine aminotransferase (IU/L)	190		60	29	26	32	40	40
Alkaline Phosphatase (IU/L)			570	177	124	136	172	136
Total Protein(g/dl)	6.0		5.0	3.9	4.2	4.3	4.3	5.8
Albumin (g/dl)	3.3		2.6	1.9	2.1	2.3	2.2	3.3
Fibrinogen(mg/dl)	80		106	123	139		158	277

**Diagnosis**: a syndromic diagnosis of acute liver failure was made. An etiological evaluation was negative for viral infection [HBsAg, IgM HBc antibody, anti-hepatitis C virus (HCV) antibody, IgM-anti-hepatitis A virus (HAV) antibody, IgM anti-hepatitis E virus (HEV) antibody], autoimmune hepatitis [anti-nuclear antibody (ANA), anti-smooth muscle antibody (SMA), anti LKM1 antibody], Wilson disease (normal ceruloplasmin and no Kayser-Fleischer ring). Among the pregnancy-related syndromes, AFLP was considered more likely than HELLP syndrome and pre-eclamptic liver dysfunction in view of encephalopathy, coagulopathy and absence of pregnancy-induced hypertension. She fulfilled 8 of 14 Swansea diagnostic criteria for AFLP, vomiting, encephalopathy, hyperbilirubinemia, transaminasemia, coagulopathy, deranged renal function, leucocytosis, ascites.

**Timeline of the current episode**: she was prescribed empirical Piperacillin-tazobactam after drawing blood cultures. Urine output and blood sugars were regularly monitored. The patient was taken up for emergency caesarean section and delivered a live low birth weight (2.4 Kg) male child. She lost 1.2 litres of blood intraoperatively and received 1 unit of packed red cells, four units each of fresh frozen plasma (FFP) and platelets, and ten units of cryoprecipitate. She was extubated in the operating room itself. She was normotensive and normoglycemic with adequate urine output during ward stay, though flapping tremors and tachycardia were persistent. Post-operative day 4 blood investigations revealed leucocytosis, persistent coagulopathy, thrombocytopenia and worsening renal dysfunction. Abdominal computed tomography scan did not reveal any collections or uterine dehiscence. In view of continued systemic inflammatory response syndrome (SIRS), antibiotics were upgraded to Meropenem and Teicoplanin. However, her initial and all subsequent blood cultures were negative.

**Therapeutic interventions**: due to persistent organ dysfunction despite termination of pregnancy, she was offered daily sessions of TPE- on postpartum days 4,5 and 6. TPE was done with centrifugal type apheresis system (Spectra Optia, Terumo BCT). In each session, 2.6 litres of plasma was removed and replaced by FFP at the rate of 45 ml/minute without periprocedural complications.

**Figure 1:**
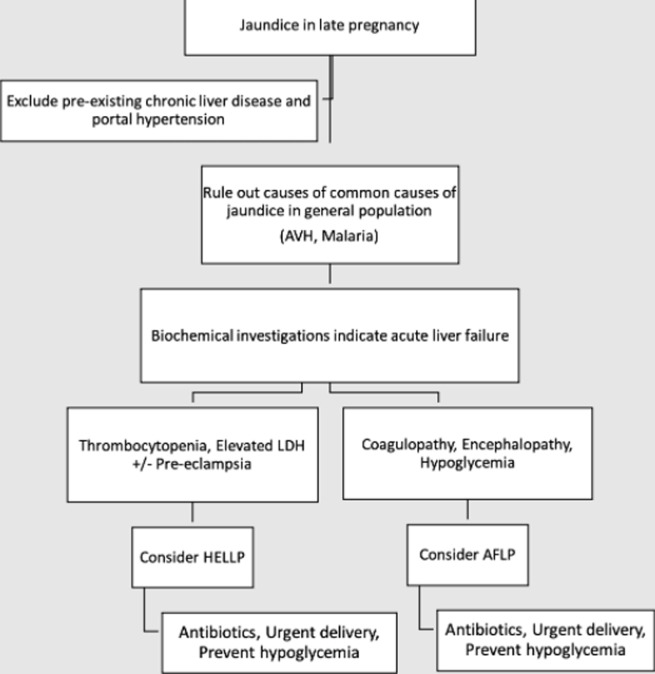
approach to liver failure in late pregnancy (adapted from Ref 2); AVH- Acute viral hepatitis; LDH Lactate dehydrogenase; AFLP Acute fatty liver of pregnancy; HELLP- Haemolysis, elevated liver enzymes, low platelets

**Follow-up and outcome of interventions**: post TPE, she had sustained clinical improvement (Figure 1), allowing tapering of antibiotics after ten days, resolution of ascites after two weeks and discharge after three weeks of hospitalization (Table 1).

## Discussion

We described an AFLP patient with persistent acute liver failure despite termination of pregnancy, where timely TPE lead to prompt recovery. AFLP is a rare hepatopathy seen in the third trimester of pregnancy, with a reported incidence of 30 cases per 1,00,000 pregnancies in India [1]. In the later stage of pregnancy (when fatty acids are the primary source of energy), foetal fatty acid oxidation defect [altered enzyme long-chain hydroxyacyl CoA dehydrogenase (LCHAD)] leads to acute fatty liver in the mother with induction of reactive oxygen species (ROS) and activation of mitochondrial caspase-3 culminating in hepatocyte apoptosis [2].

Figure 1 illustrates an approach to liver failure in late pregnancy. Emergent foetal delivery forms the cornerstone of AFLP management and, by halting worsening of liver failure, led to 96% maternal survival in a study by Nelson *et al*. [3]. In their cohort of 51 AFLP patients, 7 had hepatic encephalopathy, of which two died. Improvement in platelet count, International normalised ratio (INR), fibrinogen levels and creatinine was noted 2-3 days after delivery. In our patient, encephalopathy persisted while AKI, coagulopathy and thrombocytopenia worsened after delivery. Hence, we looked for therapeutic options to salvage the patient. The proposed mechanisms of TPE helping in hepatic recovery include removing toxic substances and supplementation of coagulation factors and albumin [4]. Tang *et al*. [2] in an in vitro study, showed that TPE induces superoxide dismutase (SOD), inhibits malondialdehyde production, protecting against oxidative damage, and inhibits upregulation of caspase-3 and caspase-9, thereby attenuating hepatocyte apoptosis.

Isolated case reports have described liver transplantation (LT) in AFLP [5]. The decision to do TPE in our patient was based on worsening clinical condition despite termination of pregnancy. Table 2 summarises existing literature on TPE in AFLP [2,4,6-10]. Similar to our observation, these reports have shown benefit of TPE in AFLP in the form of quicker normalisation of laboratory parameters, shorter hospital stay, reversal of multiorgan failure and decreased mortality. To our knowledge, this is the first report from India concerning TPE for AFLP. Larger prospective studies may better elucidate the impact of TPE on maternal survival in AFLP, but the rarity of the disease limits the feasibility of such studies. Moreover, in a resource-constrained setting such as ours, LT may not be feasible. Hence we suggest TPE as a therapeutic option to salvage AFLP patients with inadequate clinical improvement despite termination of pregnancy.

**Table 2:** summary of studies done in plasma exchange for acute fatty liver of pregnancy

Authors	Type of publication	Patients and interventions	Indication for TPE	Results
Tang *et al* [2]	Non randomised control trial	N=28 TPE, n=13 Conventional treatment , n=15 Cultured hepatocytes were treated with the plasma of patients before and after TPE and also the TPE waste replacement fluid Delivery to TPE interval= 6 hours Number of TPE sessions=1-3	Proportions of various organ failure in each group not mentioned separately	No mortality in either groups TPE group had lesser hospital stay, lower ICU stay and faster recovery of hepatic function Serum of patients in TPE group showed lower levels of Malonaldehyde (Oxidative markers), Caspase-2 and Caspase -9(apoptosis markers) after first TPE sessions compared to before TPE.
Jin *et al* [4]	Retrospective series	N=39 All underwent TPE Delivery to TPE interval= 1-5 days Number of TPE sessions=1-4	Encephalopathy (n=14) AKI (n=19) DIC (n=20)	Survival in 37(94.8%) patients Earlier initiation of TPE led to quicker recovery with lesser sessions
Martin *et al* [6]	Case series	N=6 All patients underwent TPE Delivery to TPE interval= 2-9 days Number of TPE sessions= 2-4	Liver failure, Renal failure (n=6) Respiratory failure (n=3) Circulatory failure (n=2)	Survival 100% Improvement in multiorgan failure
Tang *et al* [7]	Prospective observational study	N=11 All patients had acute kidney injury All underwent combined TPE and CVVH Delivery to TPE interval= 6 hours Number of TPE sessions=2-3	AKI (n=11) Encephalopathy (n=9) DIC (n=6)	Mortality 5.88% Higher clearance of bilirubin with TPE than CVVH
Chu *et al* [8]	Retrospective series	N=11 All patients underwent combined TPE and CHDF Delivery to TPE interval= 0-3 days Number of TPE sessions=2-8	Liver failure, renal failure (n=11) Respiratory failure (n=4)	Survival in 10 patients Resolution of multiorgan dysfunction No significant procedure related complications
Ding *et al* [9]	Retrospective study	N=22 Conventional treatment, n=16 TPE+ PP, n=6 Delivery to TPE interval= 2 weeks Number of TPE sessions=2-8	Liver failure (n=6) DIC(n=2)	83.3% survival in TPE+PP group 18% survival in conventional therapy group
Gao *et al* [10]	Retrospective cohort study	N=133 Group A= No TPE or RRT, (n=92) Group B= TPE and/or RRT, (n=41) Delivery to TPE interval= Not mentioned Number of TPE sessions=Not mentioned	Proportions of various organ failure in each group not mentioned separately	Baseline creatinine, prothrombin time and bilirubin higher in Group B Mortality Group A=12%, Group B=26.8% Bilirubin and creatinine levels independently predict mortality in Group A

## Conclusion

AFLP is a rare, life-threatening condition that responds to the termination of pregnancy in most cases. In rare instances where organ failure persists, TPE may be a readily available modality to prevent maternal mortality.
